# Evolution of the *rpoB*-*psbZ *region in fern plastid genomes: notable structural rearrangements and highly variable intergenic spacers

**DOI:** 10.1186/1471-2229-11-64

**Published:** 2011-04-13

**Authors:** Lei Gao, Yuan Zhou, Zhi-Wei Wang, Ying-Juan Su, Ting Wang

**Affiliations:** 1 CAS Key Laboratory of Plant Germplasm Enhancement and Specialty Agriculture, Wuhan Botanical Garden, Chinese Academy of Sciences, Wuhan 430074, China; 2 State Key Laboratory of Biocontrol, School of Life Sciences, Sun Yat-sen University, Guangzhou 510275, China

## Abstract

**Background:**

The *rpoB*-*psbZ *(BZ) region of some fern plastid genomes (plastomes) has been noted to go through considerable genomic changes. Unraveling its evolutionary dynamics across all fern lineages will lead to clarify the fundamental process shaping fern plastome structure and organization.

**Results:**

A total of 24 fern BZ sequences were investigated with taxon sampling covering all the extant fern orders. We found that: (i) a tree fern *Plagiogyria japonica *contained a novel gene order that can be generated from either the ancestral *Angiopteris *type or the derived *Adiantum *type via a single inversion; (ii) the *trnY*-*trnE *intergenic spacer (IGS) of the filmy fern *Vandenboschia radicans *was expanded 3-fold due to the tandem 27-bp repeats which showed strong sequence similarity with the anticodon domain of *trnY*; (iii) the *trnY*-*trnE *IGSs of two horsetail ferns *Equisetum ramosissimum *and *E. arvense *underwent an unprecedented 5-kb long expansion, more than a quarter of which was consisted of a single type of direct repeats also relevant to the *trnY *anticodon domain; and (iv) *ycf66 *has independently lost at least four times in ferns.

**Conclusions:**

Our results provided fresh insights into the evolutionary process of fern BZ regions. The intermediate BZ gene order was not detected, supporting that the *Adiantum *type was generated by two inversions occurring in pairs. The occurrence of *Vandenboschia *27-bp repeats represents the first evidence of partial tRNA gene duplication in fern plastomes. Repeats potentially forming a stem-loop structure play major roles in the expansion of the *trnY*-*trnE *IGS.

## Background

In contrast to nuclear and mitochondrial genomes, plant plastid (chloroplast) genomes (plastomes) are generally conserved in genome size, gene content and gene order [1-3]. This high conservation makes the plastid genes and genomes quite amenable for sequencing and be widely used in evolutionary and phylogenetic studies. Nevertheless, comparative genomics studies demonstrate that the plastomes of several vascular plant lineages such as lycophytes (Selaginellaceae) [4,5], gymnosperms (e.g. Pinaceae [6-8], Cupressaceae [9], Welwitschiaceae [7,10], Gnetaceae and Ephedraceae [7]) and various eudicot angiosperm lineages (e.g. Geraniaceae [2,11], Campanulaceae [12,13] and Fabaceae [14,15]), have experienced remarkable genomic changes including significant size variations, complex rearrangements as well as substantial gene losses. Many reports have shown that highly rearranged plastomes usually contain a large number of repetitive elements [2,11,12,16]. Furthermore, the distribution of the repeats also exhibits a tendency to flank the rearrangement endpoints, implying an association between the repeat and the rearrangement [2,9,11,12,16-18]. Recently, Maréchal and Brisson [19] specified that the suppression of recombination between repeats is of importance in the maintenance of plastome stability. Nevertheless, besides rearrangement endpoints, abundant repeats are also found in other regions of plastomes. For instance, extensive dispersed repeats have been found throughout the algae plastome of *Chlamydomonas reinhardtii *[20], and many direct repeats derived from partial duplication of their nearby *trnY*-GUA gene have been observed in Douglas-fir (*Pseudotsuga menziesii*) [21]. These findings highlight the structural and functional significances of chloroplast DNA (cpDNA) repeats. In *Chlamydomonas *plastomes, it has been shown that small dispersed repeats can influence both transcript stability and translation efficiency [22] or even function in DNA repair [23]. Previous studies, particularly those on the complete plastome sequences, have well documented the characteristics and distribution of cpDNA repeats [2,9,11,12,16,20,24,25]. However, very few investigations deal with the implications of the secondary structure of cpDNA repetitive elements on their origin, proliferation and potential function [26]. Delineating the secondary structural features should greatly facilitate our understanding of plastome evolution.

A number of comparative chloroplast genomic studies have uncovered structural mutations in fern (monilophyte) cpDNAs, including as many as 6 inversions and a few gene losses [24,27-32]. Specifically, one ~3.3 kb inversion (involving *trnG*-GCC to *trnT*-GGU) [27] and an inverted *trnD*-GUC gene (D inversion) [24] have been detected across ferns relative to other land plants. According to gene orders, the fern plastomes can be classified into two main types. One comprises the plastomes of taxa diversifying before the separation of the Schizaeales, which share the ancestral gene order and has been assumed to undergo no major rearrangements [33]. By contrast, the other composes the plastomes of core leptosporangiates possessing the derived gene order [33]. This derived gene order is characteristic of highly rearranged inverted repeats (IRs) with the rRNA genes arranged in reverse order in comparison to all other plants [34]. The rearranged IRs and their adjacent section of large single copy (LSC) region are thought to be generated by two partially overlapping inversions spanning LSC and IR regions [35]. Wolf *et al. *[33] recently illustrated that the two putative inversions occurred in pairs on the branch leading to the common ancestor of schizaeoid and core leptosporangiate ferns.

**Figure 1:**
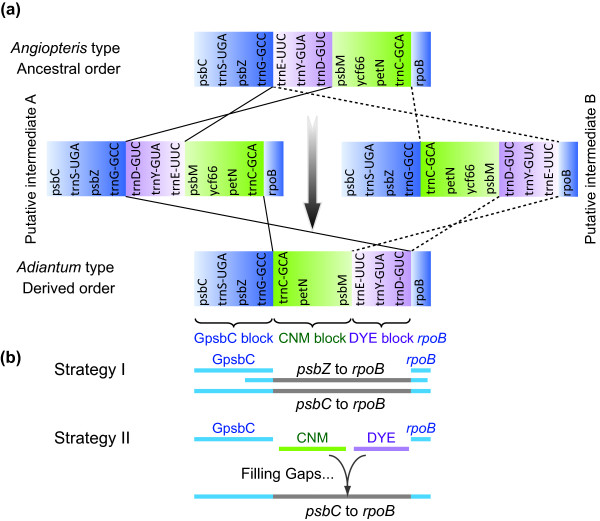
**Schematic diagrams of the fern plastid gene orders from *psbC *to *rpoB *(a) and sequencing strategies (b)**. Each colored gene segment shows the same gene order region among the published fern plastomes. The gene orders of "Putative intermediate A" and "Putative intermediate B" are according to Roper *et al. *[28].

The next striking difference between the ancestral and derived gene order is occurred between the *rpoB *and *psbZ *(BZ) in LSC region (Figure 1a). BZ region is characterized with a high degree of variability. Each of the three key inversions shaping the ancestral gene order of ferns, i.e. the 30-kb inversion [36], the 3.3-kb inversion [27] and the D inversion [24] , have at least one of their endpoints located within BZ region. Notably, up to five tRNA genes are concentrated in this small region after the three inversions (Figure 1a). This uncommonly high frequency of tRNA genes may be relative with the instability of BZ region. Roper *et al. *[28] suggested that the gene order changes within BZ region (hereafter the BZ rearrangement) of ferns can also be derived from two partially overlapping inversions by either of the two potential pathways (Figure 1a). Nonetheless, since all the investigated core leptosporangiates possess the derived BZ order (the same as *Adiantum *type gene order) (Figure 1a) and no intermediate has been identified in any ferns, it has been argued that the two hypothetical inversions should take place in pairs in the common ancestor of core leptosporangiates [33]. Unfortunately, the previous studies have only examined four complete (3 polypods and 1 tree fern) [24,27,30,32] and six partial plastome sequences from the leptosporangiates [33]. If more samples are examined, the putative intermediates may be uncovered.

In this study, we mainly investigated the evolutionary process of BZ region and its sequence components in ferns. Twenty-four fern BZ sequences were studied guided by the recently published phylogenetic framework [37], with a focus on leptosporangiates. Firstly, a novel gene order was detected in the tree fern *Plagiogyria japonica*, which may represent the intermediate of BZ rearrangement or the reverse mutant of the *Adiantum *type. Secondly, a unique 459-bp region, consisting of 17 tandem 27-bp repeats derived from the partial duplication of the adjacent *trnY *gene, was found to cause the *trnE*-*trnY *intergenic spacer (IGS) of the filmy fern *Vandenboschia radicans *to expand approximately 3-fold in length. To our knowledge this is the first report of partially duplicated tRNA gene in fern plastomes. Thirdly, unexpected 5-kb long *trnE*-*trnY *IGSs were observed in two horsetail ferns *Equisetum ramosissimum *and *E. arvense*. More than a quarter of the IGSs was comprised of a single type of direct repeats possessing the potential to form a highly conserved stem-loop structure. The direct repeats may have a recent evolutionary origin, frequently conduct copy corrections, and are of significant functional relevance. And fourthly, the occurrence of *ycf66 *was confirmed highly unstable in ferns with at least 4 times of independent losses.

## Methods

### DNA amplification and sequencing

Up to date, seven complete plastome sequences of ferns have been deposited in GenBank, whose data can be directly extracted. Besides these, additional 17 sampling taxa were chosen based on the previously published phylogenetic framework of extant ferns [37] to represent all major lineages at the order level (Table 1). Young leaves of the 17 fern species were collected from Wuhan Botanical Garden, Chinese Academy of Sciences (CAS), South China Botanical Garden, CAS, and Shenzhen Fairy Lake Botanical Garden. Voucher specimens were deposited at the herbarium of Wuhan Botanical Garden, CAS. Total DNA isolation, primer design, polymerase chain reaction (PCR) and DNA sequencing were as previously described [24].

**Table 1:** List of taxa and sequences analyzed in this study

Taxon	**Family**^**a**^	**Order**^**a**^	**Collection information**^**b**^	GenBank	Citation
*Adiantum capillus-veneris*	Pteridaceae	Polypodiales	-	NC_004766	Wolf *et al. *2003 [27]
*Asplenium australasicum*	Aspleniaceae	Polypodiales	WBG	HQ658095	This study
*Cheilanthes lindheimeri*	Pteridaceae	Polypodiales	-	NC_014592	Wolf *et al. *2010 [30]
*Platycerium bifurcatum*	Polypodiaceae	Polypodiales	WBG	HQ658094	This study
*Pteridium aquilinum*	Dennstaedtiaceae	Polypodiales	-	NC_014348	Der 2010 [32]
*Alsophila spinulosa*	Cyatheaceae	Cyatheales	-	NC_012818	Gao *et al. *2009 [24]
*Plagiogyria japonica*	Plagiogyriaceae	Cyatheales	SZBG	HQ658099	This study
*Azolla caroliniana*	Salviniaceae	Salviniales	WBG	HQ658096	This study
*Marsilea quadrifolia*	Marsileaceae	Salviniales	WBG	HQ658098	This study
*Salvinia molesta*	Salviniaceae	Salviniales	SCBG	HQ658097	This study
*Lygodium microphyllum*	Lygodiaceae	Schizaeales	SZBG	HQ658100	This study
*Dicranopteris linearis*	Gleicheniaceae	Gleicheniales	SZBG	HQ658102	This study
*Diplopterygium chinensis*	Gleicheniaceae	Gleicheniales	SZBG	HQ658103	This study
*Dipteris chinensis*	Dipteridaceae	Gleicheniales	WBG	HQ658101	This study
*Vandenboschia radicans*	Hymenophyllaceae	Hymenophyllales	WBG	HQ658104	This study
*Osmunda vachellii*	Osmundaceae	Osmundales	WBG	HQ658105	This study
*Angiopteris evecta*	Marattiaceae	Marattiales	-	NC_008829	Roper *et al. *2007 [28]
*Botrychium strictum*	Ophioglossaceae	Ophioglossales	WBG	HQ658108	This study
*Helminthostachys zeylanica*	Ophioglossaceae	Ophioglossales	SCBG	HQ658107	This study
*Ophioglossum vulgatum*	Ophioglossaceae	Ophioglossales	SZBG	HQ658106	This study
*Psilotum nudum*	Psilotaceae	Psilotales	-	NC_003386	Wakasugi *et al. *1998 [29]
*Equisetum arvense *1	Equisetaceae	Equisetales	SCBG	HQ658110	This study
*Equisetum arvense *2	Equisetaceae	Equisetales	-	GU191334	Karol *et al. *2010 [31]
*Equisetum ramosissimum*	Equisetaceae	Equisetales	SCBG	HQ658109	This study

a - the nomenclature follow Smith *et al. *[37].

b -WBG, Wuhan Botanical Garden, CAS; SCBG, South China Botanical Garden, CAS; SZBG, Shenzhen Fairy Lake Botanical Garden. All voucher specimens were deposited at the herbarium of Wuhan Botanical Garden, CAS.

To obtain the sequences from *rpoB *to *psbZ*, the conserved flanking regions, partial sequence of *rpoB *gene and GpsbC (*psbC *to *trnG*) block (Figure 1a) were amplified, cloned into plasmid vectors (pCR2.1, Invitrogen, Carlsbad, CA) and transformed into *E. coli *DH5α. At least three clones for each PCR product were randomly selected and commercially sequenced from both ends using ABI 3730xl DNA Analyzer (Applied Biosystems). Species-specific primers were then designed based on the flanking sequences and long-range PCR was performed to amplify *rpoB*-*psbZ *region (Figure 1b, Strategy I). The desired band was gel-purified, sequenced from both ends, and then determined the remains by primer walking. To avoid the potential error from PCR and sequencing, each PCR fragment was independently sequenced twice. If they had differences, additional sequencings were performed.

For some samples, whose BZ sequences were unable to be completely acquired by primer walking sequencing of PCR products because of repeats and/or complex secondary structures, a two-step approach was applied (Figure 1b, Strategy II): first, the regions of CNM (*trnC*-*petN*-*psbM*) and DYE (*trnD*-*trnY*-*trnE*) gene blocks were amplified, cloned and sequenced; second, species-specific primers were designed based on the CNM and DYE sequences coupled with the primers from the *rpoB *gene and GpsbC region to amplify the remained sections. At least three clones for each PCR product were sequenced. The overlapping regions of each pair of adjacent PCR fragments exceeded 150 bp.

The sequences generated in this paper have been deposited in GenBank (accession numbers: HQ658094-HQ658110) (Table 1).

### Sequence assembly and annotation

The individual reads were cleaned by removing vector, primer and low-quality sequences, then assembled using CAP [38] through BioEdit [39]. The assembled sequences were annotated by DOGMA (Dual Organellar GenoMe Annotator) [40]. Start and stop codons were defined through comparison to published complete plastome sequences available in GenBank. To detect tRNA genes, two online programs were employed, ARAGORN v1.2 [41] and tRNAscan-SE v.1.21 [42]. The putative promoters were identified by running BPROM [43].

### Repeat sequence analyses

The sequences were initially scanned with REPuter [44] at a repeat length ≥ 20 bp with a Hamming distance of 3. Forward (direct), reverse, complement and reverse complement repeats were all recognized under REPuter. Repeated sequences were unusually abundant in *E. ramosissimum *and *E. arvense*. For them, repeats were further identified and classified by the VMATCH software package [45]. For each sequence, an index was constructed using MKVTREE program with the -dna -pl -allout and -v options. Direct repeats ≥ 20 bp were identified using VMATCH and then divided into distinct families with MATCHCLUSTER by allowing 15% sequence dissimilarity (-erate option set to 15). The sequences of each family were extracted with VMATCHSELECT. Like REPuter, the VMATCH identifies all overlapping repeats and thus overestimates the number of repetitive elements in a given sequence. To avoid this issue, the redundant overlapping repeats were masked. The consensus for each family was then generated from a CLUSTAL X [46] alignment.

The secondary structures of repeated sequences were predicted by Mfold web server [47] with default parameters. Most of the repeats found in horsetails have a stem-loop structure with a 7-nt loop. Then, we designed a Perl script (available on request) to detect the sequence fragments which have the following stem-loop structure characteristics: loop length = 7 and stem length ≥3. The identified stem-loop sequences were assigned to distinct families according to their stem sequences afterwards.

### Phylogenetic analyses

A total of 5 protein-coding (*petN*, *psbC*, *psbM*, *psbZ*, *rpoB*) and 6 tRNA gene (*trnC-*GCA, *trnD-*GUC, *trnE-*UUC, *trnG-*GCC, *trnS-*UGA, *trnY-*GUA) sequences were extracted from 17 new generated fern plastid sequences from *psbC *to *rpoB *in this study (Figure 1). The coding sequences of these 11 genes were also acquired from the completed plastomes of 6 ferns, i.e. *Adiantum capillus-veneris*, *Alsophila spinulosa*, *Angiopteris evecta*, *Cheilanthes lindheimeri*, *Psilotum nudum *and *Pteridium aquilinum*, as well as 2 seed plant outgroups, i.e. *Amborella trichopoda *(NC_005086) and *Cycas taitungensis *(NC_009618), according to their annotations in GenBank. The nucleotide sequences of each tRNA gene were aligned in MUSCLE [48] with manual inspection. For protein-coding genes, nucleotide sequences for each gene were translated into amino acids, aligned in MUSCLE [48]. Nucleotide sequences were aligned by constraining them to the amino acid sequence alignment followed by manual adjustments. A Nexus file comprising 5,525 characters was generated after alignment was completed.

Phylogenetic analyses were performed using maximum likelihood (ML) (GARLI v1.0.699) [49] and Bayesian inference (BI) (MrBayes v3.1.2) [50]. The most appropriate model (GTR+I+G) of nucleotide evolution was determined by using the Akaike Information Criterion via Modeltest 3.7 [51]. For ML, three independent runs were conducted in GARLI, using default parameters except that automated stopping criterion set at 20,000 generations (genthreshfortopoterm = 20000). A total of 1,000 ML Bootstrap (BS) replicates was also performed using GARLI. Likelihood scores were calculated by using PAUP v4.10 [52]. For BI, each run started with a random tree, default priors and four Markov chains, and were sampled every 100 generations. Three independent analyses were run for 1 × 10^7^, 1.5 × 10^7 ^and 2 × 10^7 ^generations. Convergence was confirmed by Tracer 1.5 [53]. Twenty-five percent of burn-in trees were discarded.

## Results and Discussion

### The process of *rpoB*-*psbZ *rearrangement

**Figure 2:**
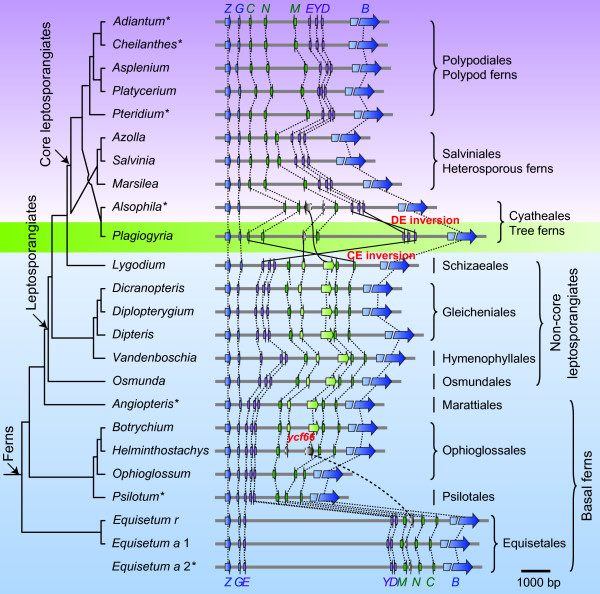
**The gene organization from *rpoB *to *psbZ *in analyzed ferns**. The arrows correlate with the location, size and transcription direction of the corresponding genes. Dashed lines indicate direction of transcription does not change; solid lines mark putative local inversions. The complete version of the tree including statistical supports and branch lengths is shown in Additional file 1. *, the plastomes have been sequenced. The symbols of *ycf66*: green, complete gene; grey, pseudogene. Abbreviations: *Z*, *psbZ*; *G*: *trnG*-GCC; *E*: *trnE*-UUC; *Y*: *trnY*-GUA; *D*, *trnD*-GUC; *M*, *psbM*; *N*, *petN*; *C*, *trnC*-GCA; *B*: *rpoB*; *Equisetum a*, *Equisetum arvense*; *Equisetum r*, *Equisetum ramosissimum*.

Two putative pathways have been proposed for describing the evolutionary process of the complex gene order change between *rpoB *and *psbZ *through fern evolution (Figure 1a) [28]. However, no direct evidence is provided for either of them. Figure 2 shows the BZ gene order in 24 samples representing all the 11 extant fern orders (Table 1) [following reference 37]. Two blocks of genes, CNM (*trnC*-*petN*-*psbM*) and DYE (*trnD*-*trnY*-*trnE*), are found to be conserved across ferns. Nearly all core leptosporangiates excluding *Plagiogyria japonica *have the same gene arrangement pattern as that observed in *Adiantum capillus-veneris *[27] (hereafter the *Adiantum *type). By contrast, all basal ferns and early branches of leptosporangiates share the gene order previously found in *Angiopteris evecta *[28] (hereafter the *Angiopteris *type). Unlike other core leptosporangiates, the tree fern *P. japonica *(Plagiogyriaceae) does not present the *Adiantum *type order. Instead its gene order (hereafter the *Plagiogyria *type) seems to derive from the *Angiopteris *type via a large inversion spanning from *trnC*-GCA to *trnE*-UUC ("CE inversion" in Figure 2) or from the *Adiantum *type through a small inversion only involving the DYE block ("DE inversion" in Figure 2).

**Figure 3:**
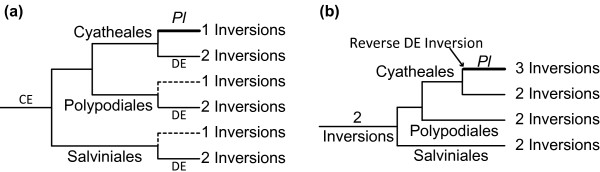
**Two potential explanations for the origin of the *Plagiogyria *gene order**. *Pl*, *Plagiogyria*. The minimal numbers of inversion events compared to the *Angiopteris *type are provided for each branch. (a), "CE" and "DE" indicate the putative CE and DE inversions as figure 2, respectively. The dashed lines show the hypothetical branches with no experimental evidence. (b), "2 inversions" denotes the two hypothetical inversions that converted the *Angiopteris *type to the *Adiantum *type [28].

The *Plagiogyria *type order seemingly represents the intermediate of BZ rearrangement. If this hypothesis is true, we might speculate that the *Adiantum *type is formed through two serial inversions, first the large CE inversion and then the small DE inversion (as shown in Figure 2). For the CE inversion, the most parsimonious explanation is that it occurred only once and on the common ancestor of core leptosporangiates (Figure 3a), because the *Adiantum *type has been observed in all the three core leptosporangiate lineages. The next question is at which evolutionary stage the DE inversion event occurred? Recent studies have identified Plagiogyriaceae as a lineage of tree ferns [54-61]. Thus, it is reasonable to expect that the *Adiantum *type found in tree ferns directly arose from the *Plagiogyria *type. As for the *Adiantum *type in other core leptosporangiate ferns, intuitively it is also intended to infer that this order was derived from the *Plagiogyria *type. However, current knowledge of the phylogenetic positions of both Plagiogyriaceae and tree ferns make the speculation implausible. Molecular phylogenetic analyses have shown that tree ferns are the sister group of polypods, and then the two groups jointly compose the sister group to heterosporous ferns (Figure 2, Additional file 1) [56,58,59,61-65]. If it is presumed that the *Adiantum *type observed in heterosporous and polypod ferns originated directly from the *Plagiogyria *type, there should exist unknown polypod and heterosporous fern species that possess the same intermediate gene order as that of *Plagiogyria*. In other words, once the *Plagiogyria *type is hypothesized to be the intermediate form of the BZ rearrangement, the putative DE inversion would have had to independently occur at least three times (each in the three core leptosporangiate lineages, respectively) to transit the *Plagiogyria *type into the *Adiantum *type (Figure 3a). Therefore, taking the *Plagiogyria *type as the intermediate form actually becomes a very unlikely pathway for establishing the derived BZ gene type.

An alternative interpretation is that the *Plagiogyria *type merely represents a derivative of the *Adiantum *type via a reverse DE inversion (Figure 3b). As shown in figure 2, the DYE block is quite short, merely ~300-500 bp in most leptosporangiates. Since it is well recognized that the small-scale inversion is highly prone to reversal and parallelism [66], and the high degree of rearrangements is often associated with tRNA genes [12], here we would propose that the occurrence of the reverse DE inversion should be of great possibility. If this is indeed the case, then the exact process of the alteration of *Angiopteris *type to *Adiantum *type remains an open question.

### *trnD*-GUC inversion

Three consecutive tRNA genes, *trnD*-GUC, *trnY*-GUA and *trnE*-UUC, are embedded in the BZ region. In seed plants, they have been shown to constitute an operon (*trnE *operon) whose transcript is processed to produce individual tRNA molecules [67]. Nevertheless, in our previous report, the *trnD *gene was found to have an opposite transcriptional direction relative to *trnY *and *trnE *in ferns based on the four completely sequenced fern plastome data available at that time [24]. With the newly determined sequences here, our previous speculation that the minor D inversion is shared by all fern lineages was further corroborated. Since the *trnD *is inverted, it is reasonable to assume that this gene is unable to be co-transcripted with *trnY *and *trnE*. In addition, the conserved "-35 box" and "-10 box" promoter sequences were also found upstream of the *trnD *gene in all the studied ferns (Additional file 2), further supporting that the transcription of the inverted *trnD *gene is independent of the *trnE *operon.

### Intergenic spacers

Sizes of the sequences between *rpoB *and *psbZ *are highly variable in ferns, ranging from 2,744 bp in *Psilotum nudum *to 7,546 bp in *E. ramosissimum*. The size variability is directly linked to the size of IGS, since both gene content and length are highly conserved in the BZ region (Figure 2).

#### The IGS of *trnY*-*trnE*

The sizes of *trnY*-*trnE *IGS (YE-IGS) are largely conservative in ferns, most of them ranging from 95 to 179 bp (Figure 2). The smallest YE-IGS, merely 16 bp, is detected in *Platycerium wallichii *(a polypod fern). In stark contrast, one filmy fern and two horsetails have experienced dramatic expansion of this region, reaching as long as 619 bp, 4,872 bp and 5,000 bp in *Vandenboschia radicans*, *E. arvense *(our sequence, hereafter *E. arvense *1) and *E. ramosissimum*, respectively. The unusual 5-kb long YE-IGS of *E. arvense *was also noted in the recently published report documenting its complete plastome sequence [[31], hereafter *E. arvense *2]. The unexpected large IGS leads us directly to the question of how the region is organized and where its component module originates from.

**Figure 4:**
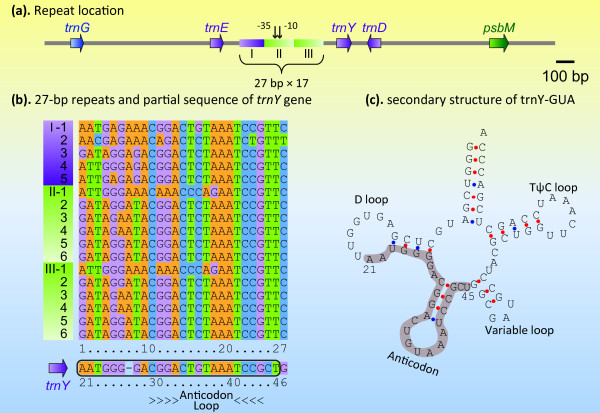
**The 27-bp quasi-identical repeats found in *Vandenboschia radicans***. "-35" and "-10" denote conserved "-35 box" and "-10 box" promoter sequences predicted by BPROM [43].

As for *V. radicans *YE-IGS, a total of 17 tandem 27-bp quasi-identical repeats were found, falling into three modules (Figure 4a). The first contains five 27-bp repeats, while the other two each include six 27-bp repeats (Figure 4b). Interestingly, the two 6 × 27 modules are identical: both are composed of one distantly homologous 27-bp head upstream of five nearly identical 27-bp segments (there is only a single base pair difference among the five repeats) (Figure 4b). We noticed that the sequences of the 27-bp repeats resemble a 25-bp section of the *trnY *gene (Figure 4b,c), corresponding to the entire anticodon arm and the stem of the D arm. Similarly, the duplications of this *trnY *region were also characterized in Douglas-fir [21]. To our knowledge, this partial tRNA gene duplication has not been reported in ferns before. Like the trnY anticodon arm, the 27-bp repetitive elements also possess the potential to fold a similar stem-loop structure. The independent occurrences of the partial *trnY *duplications in filmy fern as well as Douglas-fir imply that the anticodon domain sequence of *trnY *has a tendency to duplicate and proliferate, possibly relative to its stem-loop secondary structure.

The VMATCH software package was used to identify and classify the dispersed repeats in *Equisetum*. A total of 85 (82 direct and 3 palindromic) and 441 (440 direct and 1 palindromic) matches ≥ 20 bp were detected in the BZ sequences of *E. ramosissimum *and *E. arvense *1, respectively. All the direct matches but one from *E. ramosissimum *resides in the YE-IGS. To affirm the existence of this large number of repeats in *E. arvense*, the *E. arvense *2 plastome sequence was also analyzed by using VMATCH. 560 direct and 20 palindromic matches were recognized, of which 548 direct matches located in the YE-IGS. The YE-IGS thus far becomes the most repeat-rich region found in the *E. arvense *plastome.

After filtering the overlapping repeats, 54 and 84 non-redundant direct repeats were identified in the YE-IGS of *E. ramosissimum *and *E. arvense *1, respectively. Based on sequence similarity, the repeats fell into 16-18 families (Table 2). Their secondary structures were then predicted by using Mfold web server [47] (Additional file 3-4). Remarkably, most of the repeats, 45 out of 54 in *E. ramosissimum *and 76 of 84 in *E. arvense *1, were shown to have the potential to fold into similar stem-loop structures with a 7-nt A-rich loop and various length stem. These stem-loop repeats produce a consensus mark of three successive adenine nucleotides ("AAA") proximate to the stem (Additional file 3-4). Their total sizes are 1,154 and 2,014 bp in *E. ramosissimum *and *E. arvense *1 sequence, respectively. The uncommon abundance of the repeats implies that they may correlate to the unexpected expansion of the huge YE-IGS in *Equisetum*.

**Table 2:** Repeat families in the YE-IGS of *Equisetum ramosissimum *and *E. arvense *1 sequence identified by VMATCH

**Repeat family**^**a**^	**Consensus**^**b**^	Size	Copy Number
***Equisetum ramosissimum***			54
A	YTATGGACWWDAAATCCATAR	21	13
B	WCTGRACTCAAAATTCAGAATW	22	4
C	AAGACCTATGGACATGAAATCCATAGGTTGA	31	4
D	TAGCTRTGGACATAAAATCCATAGCT	26	4
E	TTAATTAGTTCTTGACACAAAATCAAGAACT	31	3
F	CTATGAACGTTGATAAGAACAC	22	3
G	ATTAGYTCTTGACACAAAATCRAGAA	26	3
H	ASCTMTGGACAATAAATCCATAGSTTG	27	3
I	GAATTATGGACAAGAAATCYATA	23	3
J	AGCTCTGGACATAAAATCCAGAGCTTTACGGTAG	34	2
K	ACGATCTCTGGACAAAAAATCCATAGAT	28	2
L	TTGGTGGTAAAAGCTATAGACAAGAAATCTATAGCTTG	38	2
M	TGGACTCAAAATCCATAGGTTG	22	2
N	TTTAGGTTCTTTACTTGCACTCTATA	26	2
O	TAATTAGTTCTGGACTTAAAAT	22	2
P	ATTGATTACTATATAATAAAT	21	2

***E. arvense *1**			84
A	YTATGGACAAGAAATCCATARVT	23	19
B	YTMTGGACTTAAAATCCATAGDTTK	25	17
C	TAKAWCTCTGGACTTAAAATCCATAGDTT	29	7
D	GTTTTATTTATGGACAAGAAATCCATAA	28	4
E	TTATGGACTGTAAATCCATAR	21	3
F	TTATGGACAAGAAATCCGTAACTATAGAACTAT	33	3
G	GGGTTTTATTTATGGACAAGAAATCCATAGATTG	34	3
H	TATAGTTATAGGTCTGGTGGTARA	24	3
I	TTMTGGACAASAAATCYATAAGT	23	3
J	TTGACAACAAATCCAKAATATCT	23	3
K	TAAYTTCTAGACTCAAAATCTA	22	3
L	TTTCTGGACAAGAAATCCRGAA	22	3
M	GGTACGAYTTCTGGACAATAAATCCAGAATATATGT	36	3
N	AATATCTATAGACTCCAAATCTATAGATATAGTTATAGGTTAGGT	45	2
O	TTATGGACAAGAAATCCATAAATATAGGCT	30	2
P	TTGGTGATATAACTCTGGACTTAAAATCCATAG	33	2
Q	ATATCTATAGACTCCAAATCTATA	24	2
R	ATATATGTATGGACCTGTTGACAACAAATCCATA	34	2

a - Families of non-overlapping repeats sharing ≥ 85% sequence identities;

b - The underlined DNA strings are mutually complementary and have potential to form a paired double helix. The putative secondary structures of the repeat families are provided in Additional file 3-4.

**Figure 5:**
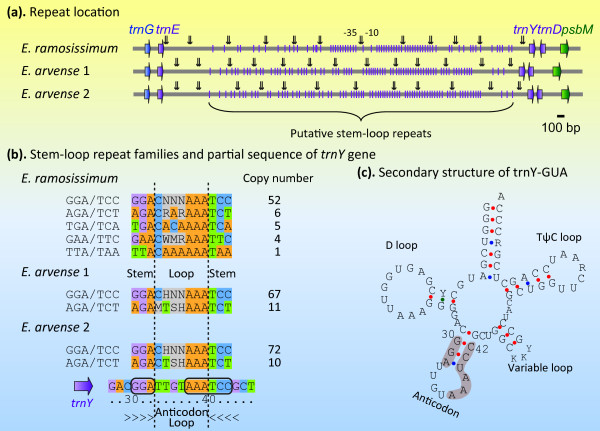
**The putative A-rich stem-loop repeats in the YE-IGS of *Equisetum***. The small black arrow pairs indicate the conserved pairs of "-35 box" (left) and "-10 box" (right) promoter sequences predicted by BPROM [43].

In order to test the correlation between the proliferation of the stem-loop sequences and the expansion of YE-IGS, we composed a Perl script to ascertain the exact amount and the distribution of the stem-loop repeats (parameters: loop size = 7, stem length ≥ 3). 90, 96 and 102 hits representing the putative stem-loop structure were identified in the YE-IGS of *E. ramosissimum*, *E. arvense *1 and 2 sequences, respectively. The majority of them, namely 68 in *E. ramosissimum*, 78 in *E. arvense *1 and 82 in *E. arvense *2 sequence (Table 3), possess the sequential "AAA" immediate to the stem (Figure 5b). The stem lengths of these A-rich stem-loop elements range from 3 to 13 bp (Table 3). It is worthy to note that the total lengths of the repeats appropriate more than one quarter of the *Equisetum *YE-IGS, i.e. 25.72%, 28.57% and 28.65% in *E. ramosissimum*, *E. arvense *1 and 2, respectively. In addition, the distribution of the stem-loop repeats is not restricted in a given small region but throughout the entire YE-IGS (Figure 5a). Our results suggest that the proliferation of the stem-loop repeats is directly correlated to the expansion of the YE-IGS in *Equisetum*.

**Table 3:** The occurrence of putative stem-loop sequences with 7-nt loop and "AAA" signature in the YE-IGS of *Equisetum ramosissimum *and *E. arvens e*

Stem length (base pair)	*Equisetum ramosissimum*	*E. arvense *1	*E. arvense *2
3	21	24	28
4	3	2	2
5	8	12	12
6	8	22	24
7	2	4	4
8	16	6	6
9	6	5	4
10	2	3	2
13	2	0	0
Total	68	78	82

The stem-loop sequences fell into 2-4 families according to the first three stem base pairs proximate to the loop (Figure 5b). The most abundant is the GGA/TCC family, which may represent the prototype of the other families. The conserved GGA/TCC stem, 7-nt loop and "AAA" signature push us to postulate that the stem-loop elements may derive from tRNA anticodon arm, because the latter often possess the same stem-loop characteristics. The complete *E. arvense *plastome sequence data shows that at least 4 tRNAs, i.e. trnC-GCA, trnF-GAA, trnL-UAA and trnY-GUA (Figure 5c), exhibit the GGA/UCC stem core, the 7-nt loop and the "AAA" signature on their anticodon regions. Of them, the *trnY *locus is exactly neighbor to the repeat region (Figure 5a). Occurrences of trnY-anticodon-arm-related repeats that are close to *trnY *gene have also been documented in Douglas-fir [21] as well as the aforementioned *Vandenboschia *(Figure 4). Taken the information together, we suggest that the *trnY*-GUA gene is possibly the origin of the stem-loop repeats, although other alternatives cannot be definitively ruled out. In contrast to the sizes and the primary sequences, the stem-loop structures of the repeats appear to be highly conservative.

The "-35 box" and "-10 box" promoter sequences were predicted upstream of *trnY *in *Vandenboschia *and *Equisetum *(Figure 4a, 5a), implying that the long YE-IGS may function in regulating the *trnY *transcription. The highly conserved stem-loop structure detected among the *Vandenboschia *and *Equisetum *repeats suggests that the repeats should potentially have a recent evolutionary origin, frequent copy corrections, and certain functional roles. Stem-loop structures have commonly been observed in the plastome IGS regions [66,68-70]. Their loop regions are often associated with hot spots for mutations, while the stem-forming sequences frequently being conserved [66]. Most plastid transcripts potentially form stem-loops in their 5' untranslated regions (5'-UTRs) and 3'-UTRs [71-73], which are thought to function in mRNA maturation, accumulation, and translation [22,71-76]. The dramatic proliferation of stem-loop repeats in the *Vandenboschia *and *Equisetum *plastomes provides a trigger for their neofunctionalization. For instance, the repeats might involve in the transcriptional and/or post-transcriptional regulation of the neighbor *trnY *gene.

#### The IGS of *psbM*-*petN *and the occurrence of *ycf66*

The other highly variable IGS is located between *psbM *and *petN *genes (MN-IGS) (Figure 2). The longest MN-IGS (1,788 bp), found in *Plagiogyria adnata*, is about 8 times longer than the shortest in *Psilotum nudum *(204 bp). Previous researches documented an open reading frame (ORF) designated *ycf66 *in the MN-IGS of *Angiopteris evecta *[28] and a pseudogenized *ycf66 *copy in both of *Alsophila spinulosa *[24] and *Equisetum arvense *[31]. Here we further identified a complete *ycf66 *in *Botrychium virginianum *(Ophioglossaceae) and all sampled "non-core" leptosporangiates (Osmundales, Hymenophyllales, Gleicheniales and Schizaeales) (Figure 2). *ycf66 *appears to be pseudogenized in *Helminthostachys zeylanica *(Ophioglossaceae), *Equisetum*, and tree ferns (Figure 2). By contrast, it was undetectable in *Ophioglossum vulgatum *(Ophioglossaceae), *Psilotum*, and polypods. Hence *ycf66 *may have been independently lost at least four times in fern lineages Ophioglossales, Psilotales, Equisetales, and core leptosporangiates. Generally, the MN-IGS containing no *ycf66 *is shorter than that carrying *ycf66 *or its pseudogene (Figure 2). For instance, of the three Ophioglossaceous ferns, the MN-IGS sizes of *Botrychium *(1,393 bp, containing intact *ycf66*) and *Helminthostachys *(1,324 bp, containing *ycf66 *pseudogene) are one time longer that of *Ophioglossum *(628 bp, containing no *ycf66*) (Figure 2). The highly unstable occurrence of *ycf66 *suggests that it seems unessential for the fern plastid function, or it has been transferred to nuclear genome.

## Conclusions

The tRNA-rich BZ region of fern plastomes exhibited considerable variation in size, gene order, and repeat content. Here a novel BZ gene order was identified in the tree fern *Plagiogyria japonica*. Our comparative analysis subsequently showed that the plastomes of extant fern lineages may not contain the putative intermediates of BZ rearrangement, pointing to the conclusion that the *Adiantum *gene order was generated by two inversions occurring in pairs [33]. The *trnY*-*trnE *IGS in the filmy fern *Vandenboschia radicans *was expanded substantially due to the tandem 27-bp repeats resembling the anticodon domain of trnY. This result provided the first evidence of partial tRNA gene duplication in fern plastomes. In general, the detection of slight length variation in chloroplast IGS region is not uncommon [e.g. [7,10,11,20]]. Nevertheless, it is unprecedented that the *Equisetum trnY*-*trnE *IGSs were found to undergo an expansion as large as 5-kb. These IGS sequences were consisted of a large amount of stem-loop repeats, which may also have an evolutionary link to the *trnY *anticodon domain. In addition, the parallel losses of *ycf66 *in ferns were corroborated.

## Abbreviations

BI: Bayesian inference; BS: Bootstrap; BZ: *rpoB *to *psbZ*; cpDNA: chloroplast DNA; D inversion, *trnD*-GUC inversion; IGS: intergenic spacer; IR: inverted repeat; LSC: large single copy; ML: maximum likelihood; ORF: open reading frame; PCR: polymerase chain reaction; plastome: plastid genome; UTR: untranslated region.

## Authors' contributions

LG conceived of the study, participated in its design, performed all sequence analyses and drafted the manuscript. YZ and ZWW participated in the sequencing and helped to draft the manuscript. YJS and TW participated in the design of the study and contributed to the interpretation of the data and prepared the manuscript. All authors read and approved the final manuscript.

## Supplementary Material

Additional file 1 **Additional figure 1**. Maximum likelihood (ML) tree of 25 taxa based on 11 plastid gene sequences Click here for file

Additional file 2 **Additional figure 2**. The predicted promoter sequences upstream of *trnD*-GUC gene Click here for file

Additional file 3 **Additional figure 3**. The putative secondary structures of the repeats found by VMATCH in *Equisetum ramosissimum* Click here for file

Additional file 4 **Additional figure 4**. The putative secondary structures of the repeats found by VMATCH in *Equisetum arvense *1 sequence Click here for file
