# Supplementary material for: Evolution of the rpoB-psbZ region in fern plastid genomes: notable structural rearrangements and highly variable intergenic spacers
Source: BMC Plant Biol. 2011 Apr 13;11:64. doi: 10.1186/1471-2229-11-64 (PMC3098776; doi:10.1186/1471-2229-11-64)
Supplement: Additional file 1 — Additional figure 1. Maximum likelihood (ML) tree of 25 taxa based on 11 plastid gene sequences [file 1471-2229-11-64-S1.PDF]

Additional figure 1. Maximum likelihood (ML) tree of 25 taxa based on 11 plastid gene sequences.

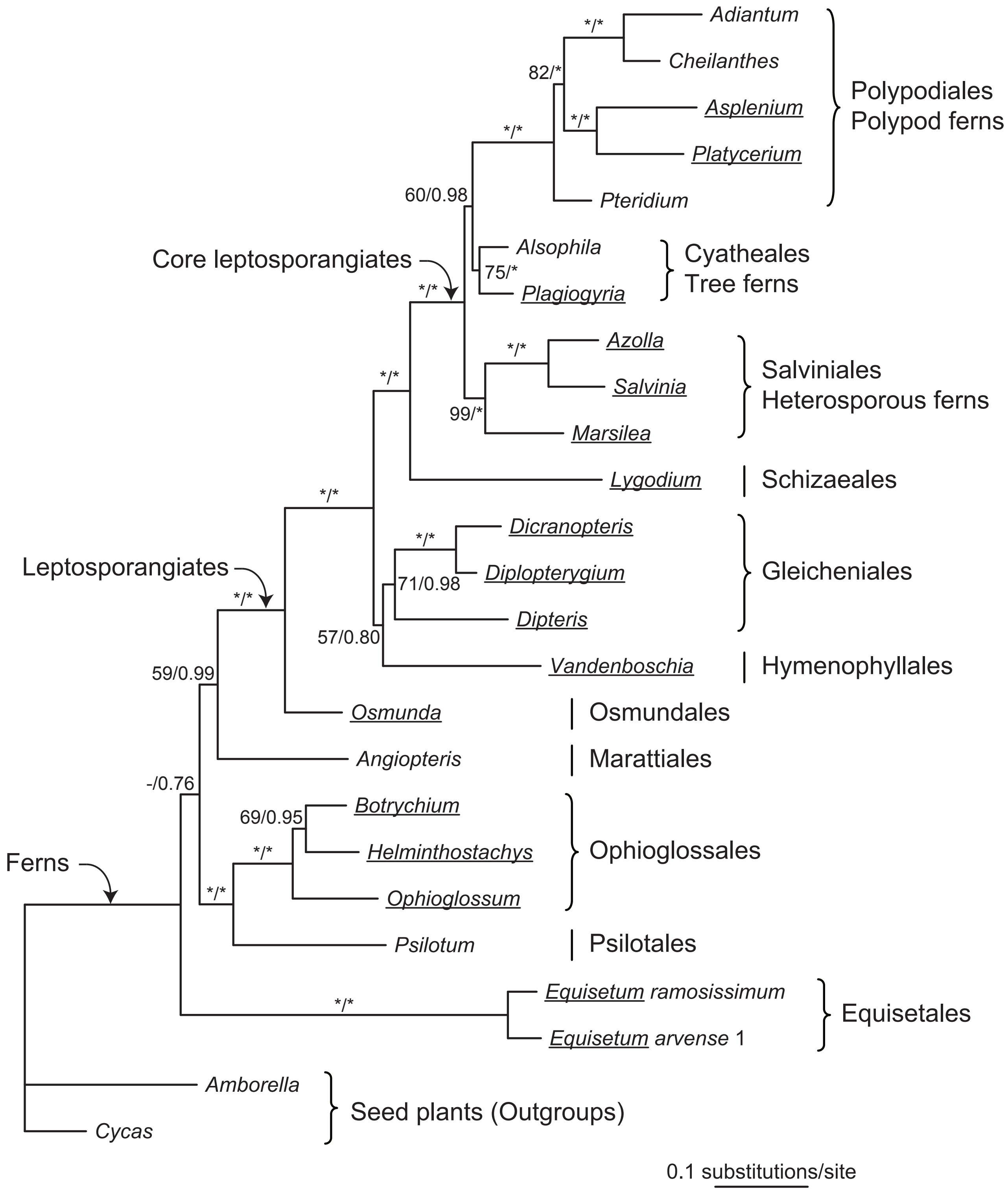

The tree has a -lnL of 47542.78697097.
Support values for ML and Bayesian inference (BI) are provided at the nodes. Asterisks indicate ML Bootstrap (BS)=100% or BI posterior probability (PP)=1.00. The hyphen (-) denotes ML BS<50%. Names for fern orders follow Smith *et al.* (2006) [37]. The tree is rooted with *Cycas* and *Amborella*. Underlined genera names indicate the sequences newly determined for this study.
