# Supplementary material for: Evolution of the rpoB-psbZ region in fern plastid genomes: notable structural rearrangements and highly variable intergenic spacers
Source: BMC Plant Biol. 2011 Apr 13;11:64. doi: 10.1186/1471-2229-11-64 (PMC3098776; doi:10.1186/1471-2229-11-64)
Supplement: Additional file 2 — Additional figure 2. The predicted promoter sequences upstream of trnD-GUC gene [file 1471-2229-11-64-S2.PDF]

Additional figure 2. The promoter sequences upstream of *trnD*-GUC gene predicted by BPROM (http://linux1.softberry.com/berry.phtml)

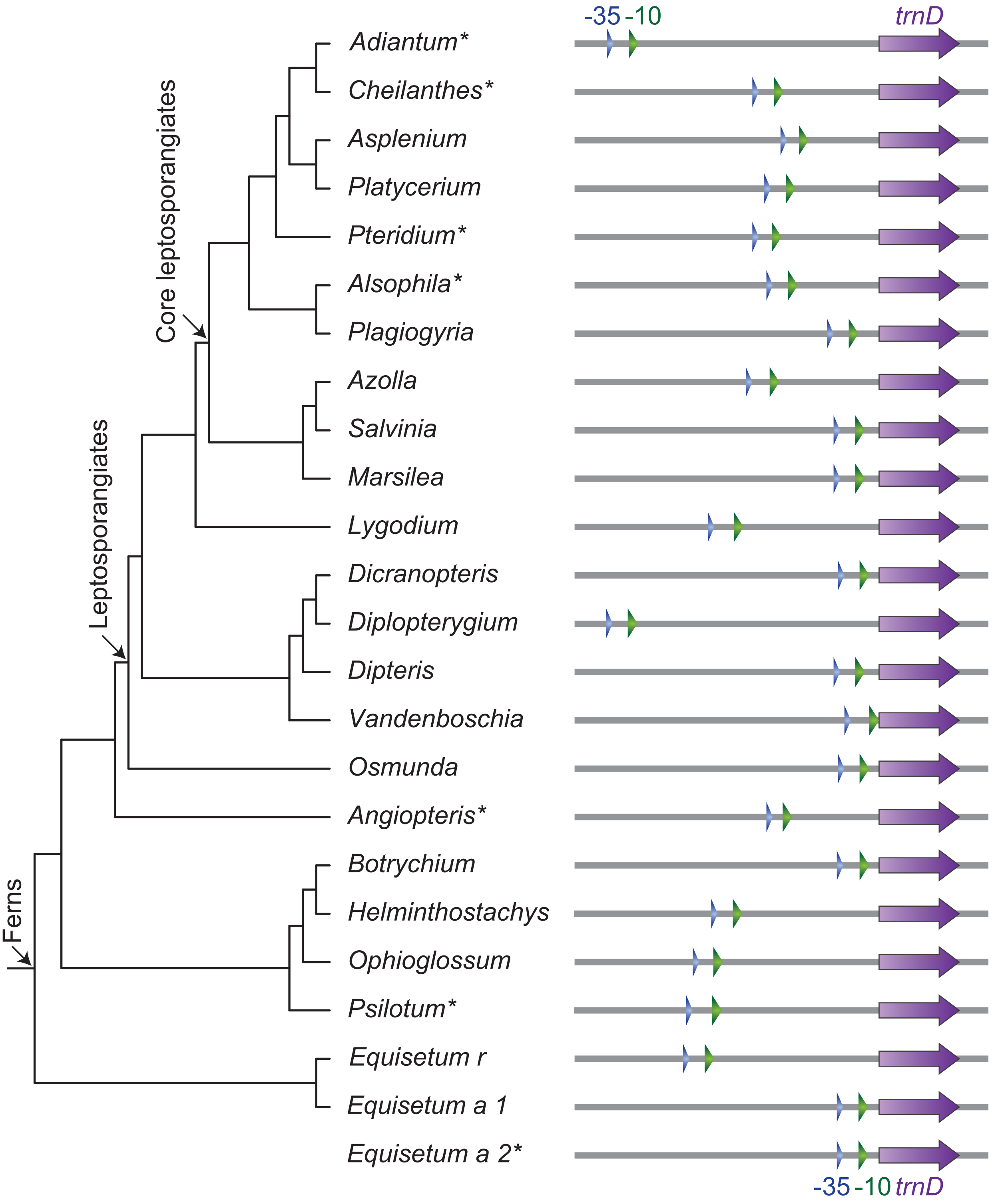

The topology of the tree is according to Additional figure 1.
