# Supplementary material for: Evolution of the rpoB-psbZ region in fern plastid genomes: notable structural rearrangements and highly variable intergenic spacers
Source: BMC Plant Biol. 2011 Apr 13;11:64. doi: 10.1186/1471-2229-11-64 (PMC3098776; doi:10.1186/1471-2229-11-64)
Supplement: Additional file 4 — Additional figure 4. The putative secondary structures of the repeats found by VMATCH in Equisetum arvense 1 sequence [file 1471-2229-11-64-S4.PDF]

Additional figure 4. The putative secondary structures of the repeats found by VMATCH in *Equisetum arvense* 1 squence

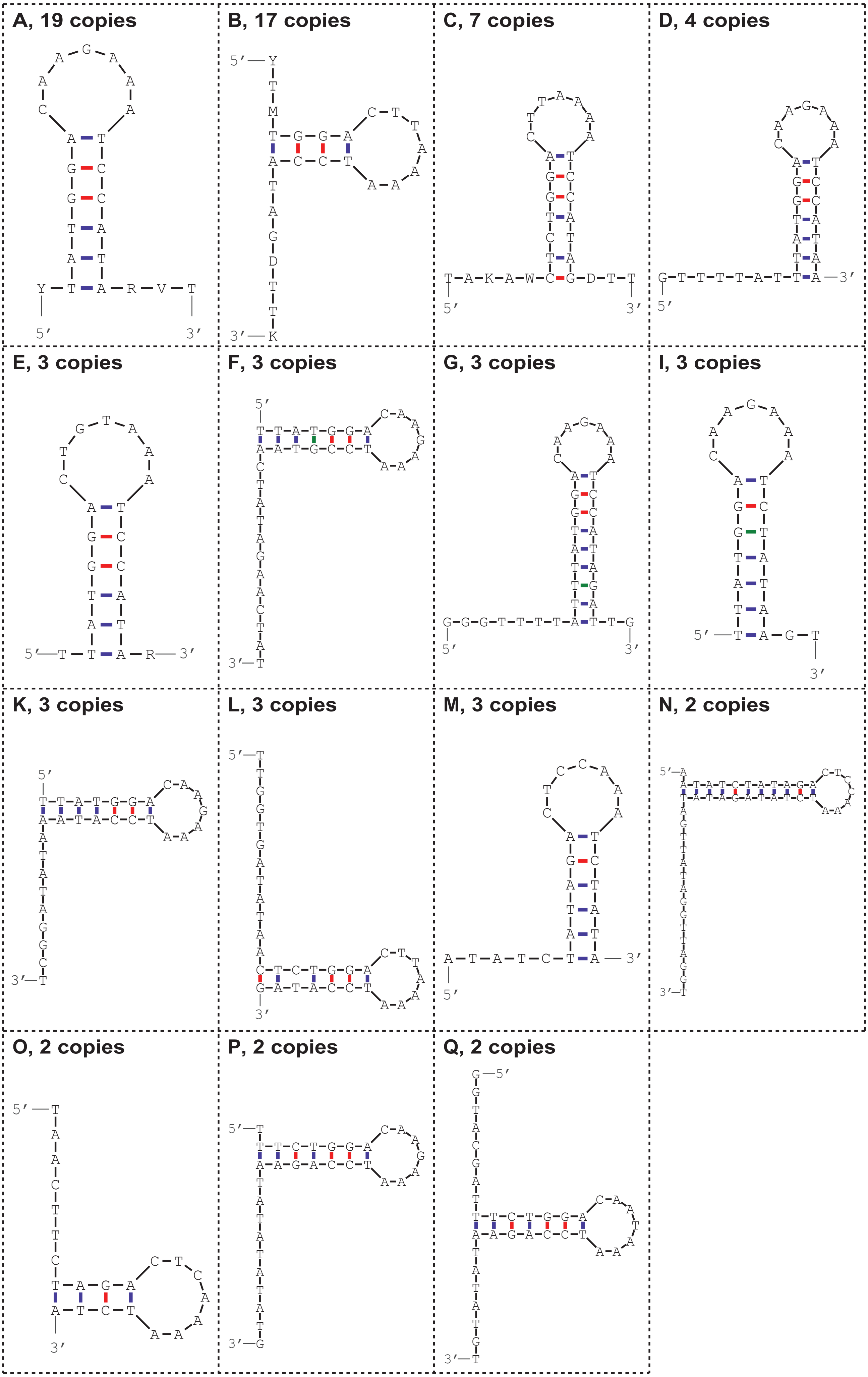

The secondary structures are predicted by Mfold web server (http://mfold.bioinfo.rpi.edu/).
